# Multi-Task Deep Learning for Joint Depth-Map Reconstruction and Iso-Point Prediction from Hyperspectral Images of Turbid Phantoms

**DOI:** 10.3390/s26206381

**Published:** 2026-10-09

**Authors:** Martin Hohmann

**Affiliations:** Institute of Photonic Technologies (LPT), Friedrich-Alexander-Universität Erlangen-Nürnberg (FAU), Konrad-Zuse-Straße 3/5, 91052 Erlangen, Germany; martin.hohmann@fau.de

**Keywords:** hyperspectral imaging, iso-point, depth reconstruction, multi-task learning, cross-attention, turbid media

## Abstract

Hyperspectral imaging (HSI) of turbid, tissue-mimicking media entangles the geometry of subsurface inclusions with the optical properties of the surrounding matrix in every spectrum. From HSI cubes of polyurethane phantoms with obliquely drilled inclusions we recover two physically distinct quantities: a dense depth map of the inclusion and the scalar iso-point wavelength, the point of near-invariance at which the depth-dependent modulation of the diffuse reflectance cancels. A single multi-task U-Net is a poor compromise for such complementary targets, the dense and scalar heads reaching their optima at different epochs while global pooling destroys the spectral-position information a wavelength target needs. We therefore decompose the problem into two specialised networks sharing one pipeline: a Bin Latent Transformer (BiLT) cross-attention scanner for the iso-point and a masked, zero-padded U-Net for the depth map. Under a strict cross-phantom split the depth network reaches a mean relative error of 6.07±0.71% ($R^{2}=0.934\pm 0.014$), against 19.4% for the analytic two-band index of earlier work and about 12.9% for the multi-task baseline. Zero-padding with a masked loss, preserving the inclusion-width information that cropping discards, is the decisive ingredient, and conditioning depth on the iso-point yields no further gain. The iso-point network reaches 5.03±0.93 nm RMSE ($R^{2}=0.630\pm 0.085$) from the spectra alone. Plausible settings of the iso-point detector change the labels themselves by 2.6 to 3.9 nm root-mean-square, while the retrained network stays between 4.8 and 5.3 nm. Together with oracle and per-patch-detection diagnostics, this identifies the ceiling as a detector-limited label-noise floor rather than a model deficiency. Averaging over a whole inclusion lowers the RMSE to a 3.27 nm plateau, separating an averageable random error from an irreducible regression-to-the-mean bias. For the straight, obliquely drilled bores tested here the depth task is solved to deployable accuracy, and the iso-point network gives a useful per-segment estimate where direct optical determination fails.

## 1. Introduction

Hyperspectral imaging (HSI) resolves the diffuse reflectance of a sample at every pixel across a continuous range of wavelengths, and in turbid, tissue-mimicking media this spectral dimension carries information about both the optical properties of the bulk material [1] and the geometry of any structure embedded beneath the surface [2]. The diffuse reflectance is governed by the absorption coefficient $\mu_{a}$ and the reduced scattering coefficient $\mu_{s}^{\prime }$ of the medium [3], optical properties whose pronounced variation between tissue types and pathological states makes them descriptors of considerable diagnostic importance [4]. A subsurface inclusion perturbs this signal in a way that depends jointly on its depth and on the contrast between inclusion and matrix. Recovering the geometry of such an inclusion, in particular its depth below the surface, is a classical inverse problem in biomedical optics: the mapping from optical properties and geometry to the measured spectrum is strongly non-linear and many-to-one, so that depth and composition are entangled in every spectrum and are difficult to disentangle by direct inversion. Model-based methods can recover depth-resolved optical properties of layered media, but rely on an explicit forward model of the light transport [5]. This motivates a data-driven approach that learns the inverse mapping from a controlled set of phantoms with known geometry, in the spirit of neural-network analyses that recover tissue optical parameters from hyperspectral skin measurements [6].

A range of optical techniques can probe the depth of structures beneath a turbid surface, but none measures depth directly: each infers it from a signal that simultaneously depends on the bulk optical properties, so depth and the underlying absorption and scattering remain confounded. Diffuse reflectance spectroscopy (DRS) sets its sampling depth through the source–detector separation (SDS), because a wider separation forces the detected photons along longer, deeper banana-shaped paths: in murine tissue at a 542 nm haemoglobin band the sampling depth grows from about 0.66 mm at a 0.75 mm separation to 1.65 mm at a 4.0 mm separation [7], while Monte-Carlo studies of layered skin report probing depths of roughly 1 to 4 mm over separations of 1.75 to 7.0 mm [8]. This accessible depth is itself entangled with the wavelength-dependent absorption, halving within the haemoglobin band at 500–600 nm relative to the neighbouring near-infrared range and depending non-monotonously on the depth of the blood-containing layer [8]. Because each spectral band therefore samples a different tissue volume, reconstruction from the full spectrum can itself introduce errors, so that quantification relies on phantom-calibrated lookup tables and several source–detector distances are needed to contrast superficial against deeper layers [7,9].

Spatial frequency domain imaging (SFDI) instead encodes depth in the spatial frequency of a projected illumination pattern, with high frequencies probing shallow and low frequencies probing deep tissue down to several millimetres in the near-infrared, where the central difficulty is decoupling the reflectance changes caused by absorption from those caused by scattering [10], a separation that recent multi-frequency, depth-resolved scattering models achieve only through an explicit layered light-transport description [11]. A complementary route, time-domain diffuse optics, resolves depth through photon time-of-flight, later-arriving photons having sampled deeper, again at the cost of specialised hardware: picosecond instrumentation, instrument-response calibration, and an explicit forward model. The depth probed in turbid media, in HSI as in all of these point and imaging techniques, changes with both wavelength and tissue composition [12]. Within HSI, this dependence has been exploited directly by selecting wavelength regions of equal imaging depth, anchored on the classical chromophore isosbestic points, to detect a tumour up to a controlled margin width [13]. These are the fixed, material-defined wavelengths at which two tissue absorbers share the same absorption coefficient, so that the imaging depth there becomes nearly independent of composition. The use of such oxygenation- and depth-invariant wavelengths to encode inclusion depth dates back to early diffuse-reflectance imaging that related them to the depth of a local blood region [14]. The depth of a subsurface inclusion has likewise been reconstructed in tissue phantoms by diffuse optical imaging, recovering three-dimensional inclusion geometry over a 1–3 mm range, yet this too relies on a forward optical model and on structured-illumination hardware rather than on the spectrum alone [15].

These chromophore isosbestic points are not to be confused with the iso-point studied here, which is not tied to any single absorber but is a depth-invariance point of the bulk diffuse reflectance whose wavelength shifts with the combined $\left(\mu_{a},\mu_{s}^{\prime }\right)$ of the phantom. The HSI margin work is nonetheless the closest published use of a wavelength of invariance to encode depth, but it rests on an explicit physical model and yields no dense depth map. Across all of these methods depth is therefore obtained indirectly and remains coupled to $\mu_{a}$ and $\mu_{s}^{\prime }$, so that disentangling the two demands forward light-transport models, calibration phantoms, or specialised hardware such as multi-distance probes, spatial modulators, or time-resolved detectors.

This coupling motivates a data-driven treatment, because the forward mapping from optical properties and geometry to the measured spectrum is strongly non-linear, ill-posed and many-to-one, the regime in which learned inverse models are most advantageous and have been surveyed as a faster, more robust alternative to forward-model fitting across diffuse optical imaging [16]. In diffuse optics, neural networks have already replaced iterative forward-model fitting for optical-property recovery: a deep network extracts SFDI optical properties several orders of magnitude faster than iterative inversion with equal or better accuracy [17], and the same speed-up now extends to U-Net architectures that map a full image to dense optical, structural and physiological fields [18]; machine learning maps diffuse reflectance directly to $\left(\mu_{a},\mu_{s}^{\prime }\right)$ without lookup tables or diffusion-model inversion [19,20], and a neural inverse model for DRS attains accuracy comparable to Monte-Carlo inversion while being validated on experimental phantoms [21], a strategy that extends directly to hyperspectral reflectance, where a physics-inspired network retrieves optical and physiological parameters at a few per cent median error while sidestepping the non-unique solutions of forward-model fitting [22]. Machine-learning-aided SFDI likewise extends optical-property recovery into the sub-diffuse regime of real tissue [23].

For the present problem, learning also recovers depth information that analytic inversion leaves averaged out: a deep network reconstructs tissue absorption and scattering in a depth-stratified rather than a bulk, depth-averaged form [24]. The depth resolution, however, still has to be supplied by an additional measurement dimension, here the photon time-of-flight of time-domain SFDI, elsewhere the source-detector separation of a probe or the spatial frequency of structured illumination, with the network only exploiting that dimension more fully.

The architecture should match the structure of each target: an encoder–decoder with skip connections such as the U-Net localises dense spatial fields precisely and trains from very few images [25], which suits a depth map, whereas attention mechanisms [26] that treat the contiguous spectrum as a sequence capture the subtle spectral discrepancies that convolutional backbones average away [27], which suits a spectral-position target such as a wavelength. The principal obstacles in this domain are the small size of calibrated medical-HSI datasets, which is partly mitigated by augmentation but encourages overfitting [28].

The two quantities extracted here from the same HSI cube differ in structure, and this difference dictates the modelling strategy: the depth map of the inclusion is a dense, per-pixel spatial field, whereas the iso-point [2] is a scalar spectral position, a single wavelength. Although multi-task models can share a single representation to ease the data scarcity that besets biomedical imaging [29], a single multi-task U-Net [25] with two heads is a poor compromise for these complementary targets, because the dense decoder and the scalar head reach their validation optima at markedly different epochs and because the global pooling feeding the scalar head destroys the spectral-position information that defines a wavelength target, the same problem solved by latent-probe cross-attention in the Bin Latent Transformer (BiLT) [30]. We therefore decompose the problem along its physics into two specialised networks that share one data pipeline: a BiLT cross-attention scanner for the iso-point and a masked, zero-padded U-Net for the depth map. We show that this decomposition closes the apparent generalisation gap of the multi-task baseline, the depth network reaching under a strict cross-phantom split an accuracy the baseline attained only under a permissive within-phantom split, with zero-padding and a masked loss preserving the inclusion-width information that cropping discards; that the comparatively low iso-point accuracy is a detector-limited label-noise floor rather than a model deficiency, established through capacity, regularisation, loss-form, oracle and per-patch-detection diagnostics; and that the iso-point network supplies a usable per-segment estimate precisely in the regime where the standard detector, which needs a wide fan of depth-varying spectra, cannot localise the iso-point at all, all benchmarked on a 238-measurement phantom dataset.

## 2. Materials and Methods

### 2.1. Phantom System and Measurements

The dataset consists of hyperspectral image (HSI) cubes of solid tissue-mimicking phantoms with three embedded inclusions (Supplementary Materials). The phantom matrix is polyurethane (PU) mixed with titanium dioxide (TiO_2_) as a scatterer and red ink (Modena Red, Montblanc International GmbH, Hamburg, Germany) as an absorber. The same ink fills the inclusions. This is a different ink from the one used in the earlier study on this phantom platform (Pelikan GmbH, Hannover, Germany) [2], so the iso-points of the two studies are not directly comparable. Both the TiO_2_ mass and the ink concentration are varied between phantoms, which spans an optical property space $\left(\mu_{a},\mu_{s}^{\prime }\right)$ that modulates the spectral response and, in particular, the position of the iso-point. The inclusion is an obliquely drilled bore. Because the bore is inclined relative to the surface, the inclusion depth varies continuously along the *y*-axis of the image; this depth gradient is the physical signal exploited by the depth-map task.

The phantom parameter grid comprises drill diameter ∈{0.6,0.8,0.9} mm, matrix ink volume ∈{60,80,100}μL, TiO_2_ mass ∈{1,2} g, and inclusion ink concentration ∈{0.05,0.25,0.5,0.75,1.0}. A complete grid would contain 3×3×2×5=90 combinations, of which 84 are realised. Including repeated inclusion locations (bottom/middle/top of the bore), the dataset contains 238 individual measurements across 84 unique phantom configurations. Each HSI cube contains 120 spectral bands spanning 400–1000 nm (≈5 nm sampling). A full hyperspectral image is taken from each phantom and cropped to each inclusion. The spatial extent of the cropped image along the *y*-axis is ≈470–490 pixels, while the inclusion width along the *x*-axis is small and varies with the drill diameter (typically 12–18 pixels).

### 2.2. Iso-Point Detection (Label Generation)

The iso-point is the wavelength at which the spectra of a phantom, measured over a range of inclusion depths, intersect with minimal spread: a point of near-invariance where the depth-dependent modulation of the diffuse reflectance cancels. It is a scalar physical property of each phantom and is used here as the regression target. The iso-point label is computed analytically from the HSI cube by a four-stage detector operating on the per-wavelength standard deviation of the spectra along a central image row, within a 550–800 nm search window. The iso-point is not a fixed wavelength. Reference [2] read it by eye at 575 nm for a single 2 mm bore, imaged from 407 to 670 nm, with the Pelikan ink as the absorber in matrix and inclusion. That study already expected the position to depend on the absorption and scattering coefficients. A different ink has a different absorption spectrum and therefore shifts the iso-point.

Prior to detection, each spectrum is vector-norm normalised along the spectral axis and denoised with a Savitzky–Golay filter [31] (window 23, order 4). The choice of the denoising filter and the refinement rule both matter: an earlier pipeline using wavelet soft-thresholding together with a high-resolution polynomial argmin refinement collapsed the iso-point distribution onto two discrete values at ≈702 and 722 nm despite the 84 distinct phantom configurations. Both steps independently quantised the detected position (soft-thresholding imprints pseudo-ripples on the scale of the wavelet support; the high-resolution argmin discretises on its sampling grid). Replacing them with the Savitzky–Golay filter and the parabolic vertex restored a continuous distribution (median per-phantom spread reduced from 19.6 to 14.4 nm). Measurements whose crossing fails the quality check are excluded from training, and no measurement of this dataset is rejected. The resulting iso-point labels span 682–738 nm across the dataset, a range of 56 nm. The value of 575 nm found in reference [2] lies inside the search window used here, and no measurement placed its crossing there. Ninety per cent of the measurements fall into the narrower window of 701–727 nm.

The detector proceeds in four stages. (i) In the candidate localisation stage, local minima of the global standard-deviation curve are extracted as crossing candidates. (ii) In the robust subset selection stage, the subset of spectra that genuinely cross at a given candidate wavelength is selected by a median-absolute-deviation (MAD)-based robust *z*-score filter, suppressing outlier spectra. (iii) In the sub-pixel refinement stage, the crossing wavelength is refined to sub-band resolution by fitting a three-point parabola to the subset standard-deviation curve and taking its analytic vertex, $\delta =\frac{1}{2}\left(y_{-1}-y_{+1}\right)/\left(y_{-1}-2y_{0}+y_{+1}\right)$. (iv) In the quality-control stage, a candidate is accepted only if the subset standard deviation shows a V-shaped divergence away from the crossing (ratio ≥1.2) together with a correlation pivot (correlation ≤0.95), ensuring a true iso-point rather than an incidental minimum.

The stability of the labels was tested by re-running the detector on all 238 measurements over a grid of its three main parameters: the Savitzky–Golay window (13, 17, 23 and 31 bands), its order (2, 3 and 4) and the MAD threshold of the subset filter (0.6, 0.8 and 1.0). For eight settings, the reference and each one-factor change along the three axes, the iso-point network was retrained over the five seeds. Its input and the phantom split were held fixed, so that only the label differs between these runs.

The iso-point is depth-dependent: across all phantoms the mean iso-point of the shallow (top) inclusion region is ${\bar{\lambda }}_{\mathrm{top}}=720.7\pm 5.4$ nm, systematically higher than that of the deep (bottom) region ${\bar{\lambda }}_{\mathrm{bottom}}=708.9\pm 8.0$ nm, with the top-to-bottom shift almost always of the same sign. For the regression target a single iso-point per phantom is used; the residual within-phantom spread after the pipeline fix (≈14 nm median) is dominated by this depth systematics rather than by detector noise.

### 2.3. Pre-Processing and Patch Extraction

The HSI cube of each measurement is divided into square patches tiled along the inclusion (*y*) axis over a contiguous span of the valid crossing region. Because the inclusion width (along *x*) varies with the drill diameter, patches are zero-padded symmetrically in *x* to a common size $X_{\max}\times X_{\max}=20\times 20$ (the next multiple of four above the largest observed width), rather than cropped to the smallest common width. Padding rather than cropping preserves the inclusion-width information that a crop would discard. For each patch the original real width $x_{\mathrm{real}}$ (number of true, non-pad pixels per row) is stored and used in two ways: (i) to build a binary pixel mask for the depth task, and (ii) to restrict the spectral feature extraction of the iso task to the true pixels. The single per-phantom iso-point label is replicated across all patches of that phantom.

### 2.4. Task Decomposition

A single multi-task U-Net with two heads (a dense depth decoder and a scalar iso regression head sharing one encoder) was used as a baseline. Diagnostics on this baseline revealed two structural obstacles to joint training: (i) the two outputs reach their validation optima at very different epochs (iso at epoch 30–60, depth at 100–150), so no single early-stopping checkpoint is optimal for both; and (ii) the global average pooling that feeds the scalar iso head collapses the spectral axis and thereby destroys the very positional information that defines a wavelength target. Therefore, the problem was decomposed along its physics into two independent networks: a Bin Latent Transformer (BiLT) [30] scanner for the iso-point and a masked U-Net for the depth map. The two networks share the same data pipeline, patch geometry, augmentation philosophy and the identical group split in every seed, but are trained and evaluated separately.

### 2.5. Iso-Point Network

The iso network is constrained to observe only the spectral content of the patch. Inclusion depth and drill diameter are, by definition, unknown quantities at inference time and are therefore not provided as inputs. Concretely, each patch is reduced to a per-wavelength spatial-statistics sequence of shape (120,7): for every band the mean, the 10th, 25th, 50th, 75th and 90th percentiles, and the standard deviation are computed over the true (non-pad) pixels only, using $x_{\mathrm{real}}$ to exclude the zero-pad region. This makes the iso network width-blind: it never sees the padding extent and therefore cannot read off the drill diameter. The real width $x_{\mathrm{real}}$ is thus used purely as a pre-processing bound (“compute statistics over the pixels that exist”), not as a feature; the same computation is valid on a real, unpadded patch. Raw per-pixel spectra were tested as an alternative input. In this variant a learned attention pooling over the true pixels replaces the seven statistics and keeps the network width-blind. A second variant admits the padded pixels and thereby exposes the inclusion width.

The sequence is processed by a BiLT scanner. The architecture is introduced in an arXiv preprint, where it serves endmember extraction for spectral unmixing [30], and it in turn adapts the latent-probe cross-attention of the Byte Latent Transformer [32]. The architecture itself is not a contribution of the present work. Only the scanner is adopted here: the physics-constrained decoder of that autoencoder is replaced by a dense regression head, and the input is an image-derived statistics sequence rather than a pair of integrating-sphere spectra. Its configuration is slimmed relative to the preprint (Table 1). A one-dimensional convolution (latent dimension 32, kernel width 15, GELU) extracts local spectral morphology in a translation-equivariant manner; a max-pooling layer adds local shift tolerance; layer normalisation and a learnable positional embedding follow. A cross-attention layer (4 learnable probe vectors as queries, 2 heads) aggregates the position-enriched feature map into a fixed-size probe representation, collapsing the spectral axis by content-weighted aggregation rather than by averaging. This is the inductive bias that preserves the positional meaning of a wavelength target while remaining robust to small spectral shifts. A self-attention block lets the four probes exchange context, followed by a feed-forward block; residual connections and layer normalisation are applied after each block. The flattened probe representation passes through a dense head (32 units, GELU, L2 $=10^{-3}$, dropout 0.4) to a single linear output. The network has ≈32,000 trainable parameters (Table 1).

A controllable spectral augmentation layer applies, to a random 50% of samples during training, a sub-band spectral shift (≤5 bands, linear interpolation) and additive Gaussian noise (σ≤0.025). Its strength is ramped in by a curriculum callback (30 clean epochs, 60 ramp epochs, full strength thereafter) to avoid destabilising early convergence. Because the spectral shift injects up to ≈15 nm of positional jitter into a positional target, the augmentation is disabled in the corresponding ablation (see the ceiling analysis in the iso-point prediction results).

### 2.6. Depth-Map Network

The depth network predicts a dense per-pixel depth map of the inclusion. Its only input is the patch cube of shape (20,20,120); like the iso network it receives no external depth or diameter label. The architecture is a two-stage U-Net [25] with skip connections, built from residual blocks [33]. The first encoder stage uses two parallel residual blocks, a spatial path (3×3 convolutions) and a spectral path (1×1 convolutions), fused by addition, so that spatial and spectral information remain separable. A second residual stage, a bottleneck of two 3×3 convolutions with spatial dropout, and a symmetric decoder with skip connections to the two encoder stages complete the network. The output is a 1×1 convolution with linear activation. Stochastic-depth regularisation is applied inside the residual blocks (Table 2).

Zero-padding introduces up to ≈40% pad pixels per patch. Trained naïvely, the network would primarily learn to predict zero in the pad region, diluting the gradient on the true pixels, and relative error metrics would diverge at the trivially zero pad pixels. The depth target is therefore packed as a two-channel array (channel 0: depth, channel 1: binary real-pixel mask) and trained with a masked mean-squared error,

(1) $$L_{\mathrm{depth}}=\frac{\sum_{p}{\left(d_{p}-{\hat{d}}_{p}\right)}^{2}\;m_{p}}{\sum_{p}m_{p}},$$

where $m_{p}\in \left\{0,1\right\}$ is the mask, so that the gradient exists only at true pixels. The corresponding masked $R^{2}$ metric and all reported depth metrics are likewise computed over true pixels only. Geometric augmentations (horizontal/vertical flips, $90^{\circ }$ rotations) are applied synchronously to the input cube and to the two-channel target so that depth and mask transform together; additive input noise is never applied to the labels.

### 2.7. Training

Both networks use the AdamW optimiser [34] with a cosine learning-rate decay [35] and a batch size of 64, implemented in TensorFlow/Keras [36]. The iso network is trained for up to 350 epochs (cosine decay $5\times 10^{-4}\to 10^{-6}$) with the delayed augmentation ramp-up and early stopping on the validation loss (patience 180, starting from epoch 90). The depth network is trained for up to 200 epochs (cosine decay $4\times 10^{-4}\to 10^{-6}$) with early stopping on the masked validation loss (patience 120). Inputs and targets are scaled by scalers fitted on the training split only (per-channel standardisation of the iso features, min–max scaling of the iso label, separate min–max scaling for the depth cube and map) and inverse-transformed for evaluation. For the iso loss, the Huber loss (δ=0.1 in the scaled [0,1] label space, ≈4 nm) [37] is used.

### 2.8. Evaluation Protocol

Generalisation is assessed under a strict group (cross-phantom) split: all patches of a given phantom are assigned entirely to either the training or the test set, so that the test phantoms are never seen during training (75 train/9 test phantoms per seed). The split is drawn once per seed and shared by both networks. They are trained on the same training phantoms and validated on the same validation phantoms. Both are evaluated on the same nine test phantoms, so their seed-wise metrics are directly comparable. This is contrasted with a random split, in which patches of the same phantom may appear on both sides. The random split measures only within-phantom interpolation: a scalar iso head can memorise a phantom from a few of its patches and trivially predict the rest, so the random split overstates the achievable cross-phantom accuracy by roughly a factor of two and is reported only as a within-phantom reference. All paper-relevant claims use the group split.

Because the training stochasticity (≈14% variation in iso RMSE at fixed configuration) exceeds the effect of most architectural levers, every configuration is run as a multi-seed sweep (n=5, seeds {0,1,2,3,42}) and reported as mean ± standard deviation. With only 9 test phantoms per seed, a single off-grid test phantom can dominate a seed’s metric, so the seed spread reflects test-set composition in addition to optimisation noise.

As a baseline from prior work, the two-band depth index of reference [2] is evaluated on the same test phantoms. It is defined per pixel as $d=1-I\left(\lambda_{\mathrm{iso}}-\Delta \right)/I\left(\lambda_{\mathrm{iso}}\right)$ and was introduced for a single phantom with $\lambda_{\mathrm{iso}}=575$ nm and Δ=25 nm. The index is dimensionless and is converted into depth by a polynomial calibration against the ground-truth depth maps. Because the iso-point varies between the present configurations, the free parameters are selected per seed on the validation phantoms: the offset Δ (10 to 80 nm), the iso-point (detected per measurement or the training median), a linear or quadratic calibration and an optional 5×5 median filter. The selected variant is calibrated on all training phantoms and evaluated on the test phantoms with the masked metric of the depth network. As the most favourable case, the index is also calibrated separately for each phantom on its own pixels, as in reference [2].

## 3. Results and Discussion

### 3.1. Iso-Point Prediction

Table 3 reports the iso-point accuracy under the cross-phantom group split. The task-decomposed BiLT scanner reaches an iso-point RMSE of 5.03±0.93 nm and $R^{2}=0.630\pm 0.085$ (n=5), against an iso-point range of 56 nm. The seed-to-seed spread reflects the test-set composition noted above: the weakest seeds are those whose nine test phantoms include an example near the extreme of the iso-point range. The training and validation loss curves no longer diverge (Figure 1), and the predicted-versus-true scatter (Figure 2, top row) is continuously populated along the diagonal across the full ≈695–735 nm range with no residual bimodal clustering, confirming that the corrected label pipeline turned what had appeared to be a quasi-two-class problem into a genuine continuous regression.

The accuracy floor does not depend on the detector setting as long as the labels remain plausible (Table 4). For the reference, both alternative orders, both alternative MAD thresholds and the wider window, the retrained network reaches 4.84 to 5.33 nm RMSE and $R^{2}$ of 0.60 to 0.69, within one seed standard deviation of each other. Only the two narrow windows at order 4 raise the error, to 6.95 and 7.73 nm. These settings smooth least, and their labels deviate from the reference by 5.0 and 7.4 nm root-mean-square. The network error rises with this added label noise, whereas against the reference labels the networks of all eight settings lie between 4.84 and 5.51 nm. Between plausible settings that change the window or the order, the labels themselves differ by a root-mean-square of 2.6 to 3.9 nm. This is of the same order as the network error, so several nanometres of that error are set by the definition of the label.

The iso-point $R^{2}\approx 0.63$ is well below the depth $R^{2}$ (≈0.93), and we trace its origin through a series of controlled experiments (Table 3 and per-phantom diagnostics). Aggregating predictions per phantom shows that patches of a single phantom predict consistently (within-phantom prediction spread ≈5 nm), but with a phantom-specific bias of up to ±16 nm against the true value (Figure 3). The bias varies linearly with the true iso-point: positive at low iso-points (≈700 nm), negative at high iso-points (≈725–738 nm), crossing zero near the training mean (≈715 nm). This is the textbook signature of regression-to-the-mean under a squared-error objective on a small sample, and it is the dominant error mechanism behind the $R^{2}$ ceiling. Narrow inclusions (D=0.6 mm) are systematically harder (|bias| ≈8 nm vs. ≈5 nm at D=0.8/0.9 mm), consistent with fewer true pixels feeding the per-wavelength statistics and hence higher feature variance; the other phantom parameters show no systematic bias trend.

Two error components, an averageable random part and an irreducible systematic part, can be separated directly by averaging the per-patch predictions. Averaging the predictions of *k* patches of the same phantom and re-computing the metrics at each *k* (Figure 4) interpolates between the per-patch estimate (k=1, reproducing the ≈5.0 nm/$R^{2}\approx 0.63$ of Table 3) and a single whole-bore estimate (one prediction per inclusion, *k* equal to all of its patches). Whole-bore averaging lowers the RMSE to a plateau of 3.27 nm and raises $R^{2}$ to ≈0.71, and no further. The ≈5.0 →3.27 nm drop is the averageable random patch-level noise; the 3.27 nm plateau is the systematic per-phantom bias that no amount of averaging removes, the same regression-to-the-mean offset seen in Figure 3. In these terms, the averageable random component accounts for about a third of the single-patch RMSE (the 5.0→3.27 nm reduction) and roughly half of its error variance, while the remaining, data-conditioned systematic part cannot be averaged away. In deployment, where all patches of an inclusion are available, the relevant per-inclusion error is therefore the plateau value rather than the single-patch figure, but the systematic floor itself persists.

The averageable component has an identifiable source. Within one bore the mean depths of the individual patches scatter with a median standard deviation of 0.97 mm, whereas the depth blur inside a single patch is 0.06 mm. The iso-point falls with depth at $4.48\;\pm \;0.31\;\mathrm{nm}\;{\mathrm{mm}}^{-1}$, from a pooled linear fit across the repeated bore locations of each configuration (235 measurements, 81 configurations). The constant per-phantom label therefore misses the true per-patch iso-point by about 4.3 nm, against 0.26 nm of in-patch blur. This is of the same order as the 3.8 nm averageable component that Figure 4 isolates in quadrature ($\sqrt{5.03^{2}-3.27^{2}}$), so the replication of a single label across patches is consistent with being its dominant source. The 3.27 nm plateau is not explained by it, since a per-phantom offset survives any per-patch relabelling.

The training-loss floor (≈0.004) is invariant across four independent interventions: doubling the model capacity (latent 64/8 probes), reducing the regularisation (dropout and L2 down), disabling the spectral augmentation, and changing the loss form (MSE, pinball and Huber differ in central value by less than the seed spread). The loss form does not move the mean; only the Huber loss reduces the seed spread (−18% in $R^{2}$ std), making it the most robust estimator without significantly improving the central value. Replacing the seven statistics by raw per-pixel spectra leaves the RMSE unchanged within the seed spread (5.06±0.93 against 4.97±0.78 nm in the same re-run, n=5). Admitting the padded pixels, which exposes the inclusion width, gives 5.07±1.12 nm. The ceiling is thus a property of the data, not of the model or the optimiser.

To split the remaining error into (a) label noise and (b) feature insufficiency, the true optical-property drivers were supplied as auxiliary inputs, a diagnostic upper bound on what perfect knowledge could achieve, not a deployable model (the concentrations are unknown in use). Even a maximal oracle (true absorber/scatterer drivers and inclusion geometry: ink concentration, TiO_2_ mass, matrix ink volume, drill diameter) lifts the result only to $R^{2}=0.703\pm 0.065$/RMSE 4.52±0.90 nm (Table 3), up from the spectrum-only default (0.630±0.085/5.03 nm). The lift (≈+0.07 in $R^{2}$, −0.5 nm) is small relative to the seed spread, so the ceiling is set predominantly by (a) irreducible label noise rather than by (b) feature insufficiency: a hard, data-side bound near $R^{2}\approx 0.70$ that even perfect knowledge of the constituents barely exceeds.

This label noise can be traced one level deeper, to the iso-point detector itself. By construction the detector localises the iso-point as the wavelength at which a fan of depth-varying spectra crosses with minimal spread, so a clean, well-localised crossing requires a wide span of inclusion depths to be present in the spectra that are pooled. On the scale of a single patch (≈20 px along the bore, a narrow depth span) this requirement is no longer met and the detector breaks down: a training-free diagnostic that re-runs the detector per patch yields a residual scatter of 65–80 nm, more than an order of magnitude above the ≈5 nm RMSE ceiling, and supplying 15× more pixel samples reduces it only marginally (80→65 nm). The per-patch label noise is therefore set by the detection geometry, not by the sample count. Crucially, this is a limitation of the label-generation method, not evidence that the per-patch information is absent: the inclusion depth map shows a well-defined between-patch depth variation within each phantom (about 4.3 nm iso-equivalent, against 0.26 nm of in-patch blur; see above), so a depth-driven per-patch iso-point signal does exist geometrically; it simply cannot be recovered by std-crossing detection on a single patch, and the single per-phantom label used here necessarily averages it away. The iso-point ceiling is thus a detector-limited label-noise floor, not a fundamental absence of spectral signal and not a model deficiency.

Two practical points follow. First, the network does provide a usable iso-point estimate from the spectra alone for an individual bore segment, a regime in which a direct optical determination is difficult, since the standard detector itself needs a wide fan of depth-varying spectra and cannot localise the iso-point on a single patch at all. A per-segment estimate of ≈5 nm, and 3.27 nm once the per-segment estimates are averaged over a whole inclusion (Figure 4), is a useful answer where the alternative is none. Second, and conversely, we stress that the reference iso-points are themselves only approximate: the standard detection is noisy, and the measured spectra carry their own noise, so both the training targets and the evaluation ground truth are imperfect. The single-patch error must therefore be read against an inherently noisy label rather than against an exact reference, and the accuracy is bounded by how precisely the iso-point can be determined from these phantoms at all, not by the predictor (capacity, regularisation, loss form, and even oracle knowledge of the optical-property drivers were all exhausted above).

### 3.2. Depth-Map Prediction

Under the same cross-phantom split, the masked U-Net predicts the inclusion depth map with a mean relative error of 6.07±0.71% and $R^{2}=0.934\pm 0.014$ (masked, over true pixels only; masked MSE 0.183±0.039 in scaled units). This more than halves the depth error of the multi-task baseline (≈12.9% MRE) and reduces the $R^{2}$ spread threefold. Notably, the cross-phantom depth accuracy now matches, indeed slightly surpasses, the random-split (within-phantom) accuracy of the baseline (≈6.7% MRE): the apparent generalisation gap of the baseline was not a fundamental cross-phantom limit but an artefact of joint training and of cropping away the inclusion-width information. Zero-padding with a masked loss preserves that width information and is the decisive ingredient for the improvement (Figure 5). For the geometry tested here, straight bores of constant width producing a smooth, monotone depth gradient in a homogeneous matrix, the depth task is solved; the remaining analysis concerns the iso-point.

The analytic two-band index of reference [2] is not competitive. Calibrated on the training phantoms, it reaches a masked MRE of 19.4±1.2% and $R^{2}=0.30\pm 0.07$ on the same test phantoms, against 27.1% for a constant prediction of the mean training depth. Even when calibrated separately for each phantom on its own pixels, the most favourable case, it reaches only 15.6 to 15.9% and $R^{2}$ of 0.53 to 0.55 for the selected variants. Within one phantom the index correlates with depth at a median of only r=−0.6. Reference [2] reported the method to work up to about 4 to 6 mm, whereas the present bores span 2.6 to 11.1 mm. The method also requires the iso-point as an input, which the detector can only determine from the depth-varying spectra of the same measurement.

The mean error hides a tail, which Figure 6 resolves. The distribution is taken from a re-run of the depth network on the identical five splits that stores every test prediction (5.85±0.54% MRE, within the seed spread of the value above). Pooled over the five seeds, the per-patch masked relative error has a median of 4.0%, a 95th percentile of 17.3% and a maximum of 48.5%. Per phantom the median is 5.4%, and the worst of the 45 test phantoms reaches 12.1%. The tail is carried by the narrowest bore: patches with a drill diameter of 0.6 mm have a median of 6.4% and a 95th percentile of 24.7%, against 3.2 to 3.8% and 12 to 13% for the wider bores. This is consistent with fewer true pixels per patch. Pixels shallower than 3 mm reach a median of 12.3%, because the relative error divides by a small depth, but they form only 1.4% of the true pixels. The worst patch and the worst phantom belong to the same configuration, a 0.6 mm bore with the lowest ink concentration in the inclusion.

A final ablation confirms that the two tasks are genuinely separable and that nothing is lost by training them apart. One might expect the iso-point, as a scalar summary of the bulk optical properties, to help the depth reconstruction, since absorption and scattering also set the contrast through which depth is read. We tested this directly by conditioning the depth network on the iso-point through feature-wise linear modulation (FiLM): a learned affine transform of the bottleneck features whose scale and shift are produced from the iso-point scalar, initialised so that the conditioned network starts exactly at the unconditioned baseline. To obtain an upper bound on the possible benefit we fed the network the true iso-point (an oracle), and we also tested the deployable variant in which a jointly trained head supplies a predicted iso-point. In neither case did the depth accuracy improve beyond the seed spread of the unconditioned baseline (MRE ≈4.7–6.8% across seeds, $R^{2}\approx 0.93$; Figure 7), and this held both for the raw FiLM and for a tanh-bounded, L2-regularised version. No feature visualisation of the bottleneck was performed, so the reason for this result is not established. It is assumed that the U-Net already extracts the optical-property information that the iso-point summarises directly from the full spectral cube, in which case the scalar is not new information for the depth task. A second consideration concerns what the scalar could correct at all: it is constant across the patches of a phantom and can therefore only remove a per-phantom offset in the depth error. Such an offset exists and carries about a quarter of the squared depth error in the re-run of Figure 6, but it is uncorrelated with the iso-point (r=0.10, p=0.51, 45 test phantoms), so the iso-point cannot predict it. Probing the bottleneck features against the known matrix parameters would test the first explanation, and it is left open. The measured independence is itself the empirical justification for the full decomposition, since the cleaner inductive bias of two specialised networks costs no cross-task synergy.

The phantoms tested here are deliberately simple: straight bores of constant width in a matrix that is homogeneous within one phantom. The aim of this study is to establish what can be recovered in that controlled case, not to demonstrate transfer to tissue, and the results are not expected to carry over one to one. The two parts of the approach transfer differently. The depth network holds no geometric prior beyond the patch shape, so a curved or branching inclusion is a question of training data rather than of architecture, which this dataset cannot test. The iso-point label is the more restrictive part, because its detector requires a fan of spectra that differ in depth and in nothing else. In a heterogeneous medium the spectra differ in optical properties as well, and a single iso-point per region loses its meaning once the bulk properties vary inside that region.

## 4. Conclusions

We have shown that two physically distinct quantities, the dense depth map of a subsurface inclusion and the scalar iso-point wavelength, can be recovered from hyperspectral images of turbid phantoms by decomposing the problem along its physics into two specialised networks rather than forcing a single multi-task compromise. The masked, zero-padded U-Net reconstructs the inclusion depth map with a cross-phantom mean relative error of 6.07±0.71% and $R^{2}=0.934\pm 0.014$, more than halving the multi-task baseline and reaching, under the honest group split, the accuracy that baseline attained only under a permissive within-phantom split; preserving the inclusion-width information through padding and a masked loss, rather than cropping it away, is the decisive ingredient. For the straight bores of constant width tested here, the depth task is thereby solved to deployable accuracy. A control experiment showed that conditioning it on the iso-point, even on the true iso-point, yields no further gain, which indicates that the two tasks share no exploitable information and supports their separation.

The iso-point network reaches 5.03±0.93 nm RMSE ($R^{2}=0.630\pm 0.085$) from the spectra alone, and a sequence of controlled diagnostics located the origin of this lower ceiling. It is not a model or optimisation deficiency: capacity, regularisation, loss form, and even an optical-property oracle all leave the ceiling essentially unchanged. It is instead a detector-limited label-noise floor. The standard iso-point detector localises the crossing from a wide fan of depth-varying spectra and cannot resolve it on the scale of a single patch, so the per-phantom labels are intrinsically noisy; the residual per-patch error separates into an averageable random part, which whole-bore averaging removes down to a 3.27 nm plateau, and an irreducible systematic per-phantom bias with the textbook signature of regression-to-the-mean. Importantly, this is a limitation of the label-generation method and not evidence that a finer signal is absent: the depth map shows a well-defined between-patch depth variation that no per-phantom label can express, which points to the most promising direction for future work. Depth-anchored iso-point targets, derived from the ground-truth depth map rather than from per-patch detection while keeping depth out of the network input, together with richer spectral features and targeted data acquisition at the extremes of the iso-point range, are the natural next levers for raising the iso-point accuracy beyond a detector-limited floor.

Three results reach beyond this phantom series. First, on the same test phantoms the network lowers the depth error of the analytic two-band method of the earlier study [2] from 19.4 to 6.07%. The two-band index correlates only weakly with depth inside one phantom, whereas the network reads depth from the full spectral cube. Second, the iso-point is not a fixed wavelength. It lay at 575 nm with the earlier ink and lies at 682 to 738 nm with the present one. An iso-point method therefore has to determine it for each sample, and the network does so from the spectra alone. Third, plausible detector settings change the labels by 2.6 to 3.9 nm root-mean-square. An accuracy reported for iso-point prediction can therefore only be read together with the uncertainty of its labels. For the geometry tested here, the framework delivers depth maps at about 6% relative error and a per-segment iso-point estimate in the regime where direct optical determination fails.

## Figures and Tables

**Figure 1:**
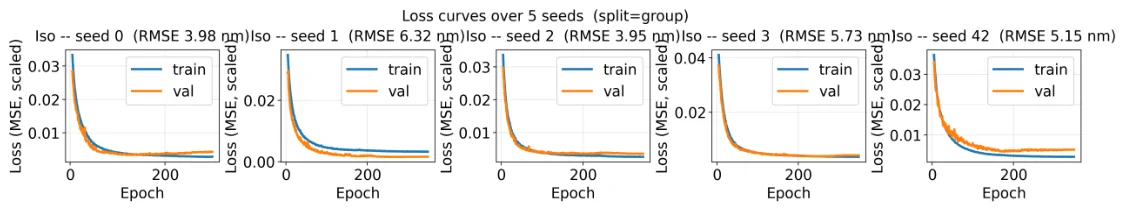
Training (blue) and validation (orange) loss for the iso-point network across the five seeds. The curves converge without a persistent train/validation gap, the validation loss tracking the training loss closely throughout; the per-seed epoch count differs because of early stopping.

**Figure 2:**
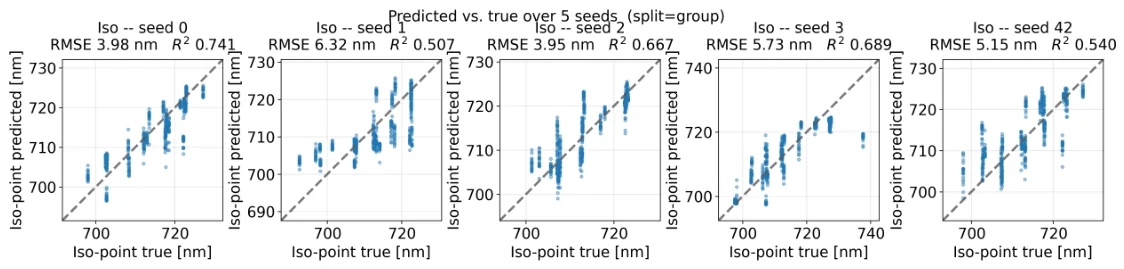
Predicted versus true values per seed (dashed line: identity). Top row: iso-point per patch, continuously populated across the label range; the vertical clustering is the per-phantom signature, where patches of one phantom share a near-constant prediction (cf. Figure 3). Bottom row: depth per masked pixel, densely scattered along the diagonal.

**Figure 3:**
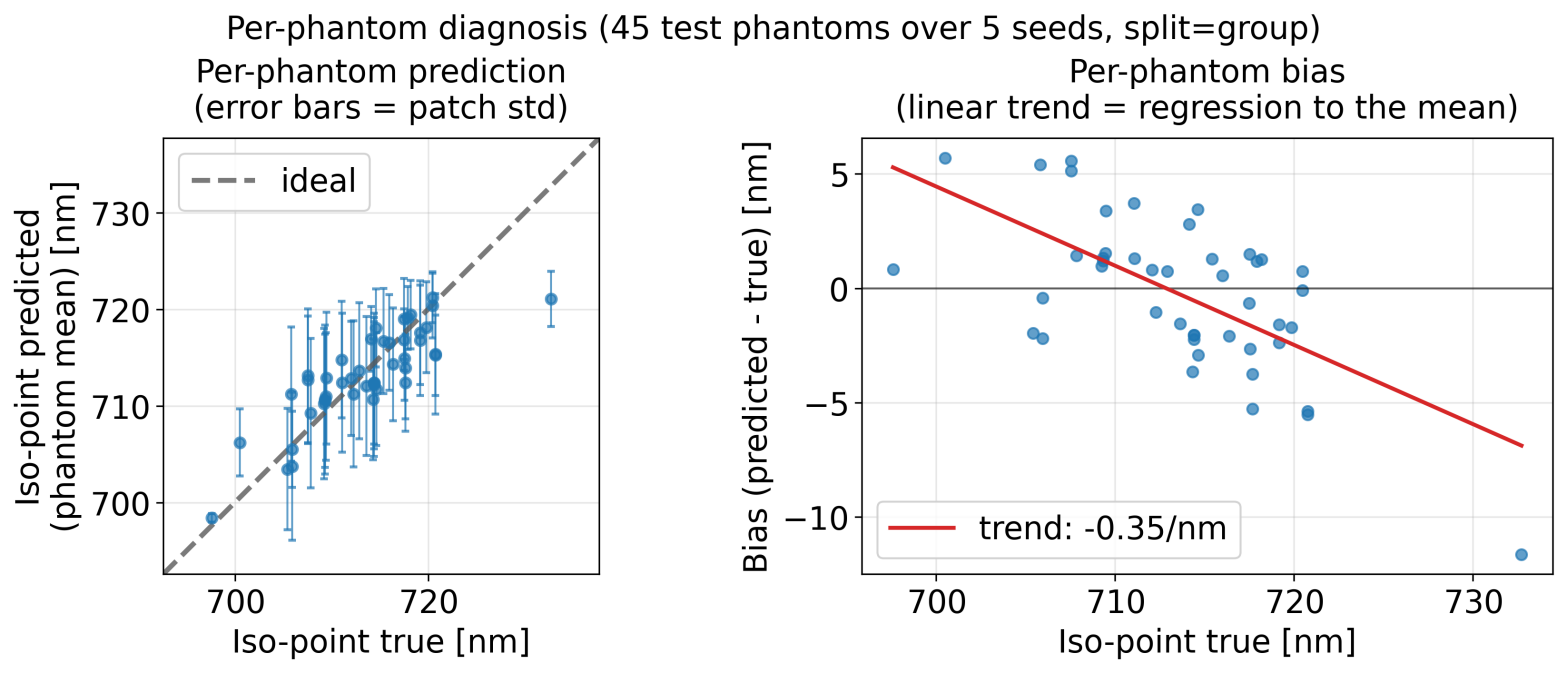
Per-phantom diagnosis pooled over all seeds (45 test phantoms). **Left**: per-phantom mean prediction (error bars: within-phantom patch spread) versus true iso-point; the error bars are small relative to the scatter about the identity line, so the residual is a per-phantom offset rather than patch noise. **Right**: per-phantom bias versus true iso-point with a negative linear trend (≈−0.2 nm per nm), the regression-to-the-mean signature underlying the $R^{2}$ ceiling.

**Figure 4:**
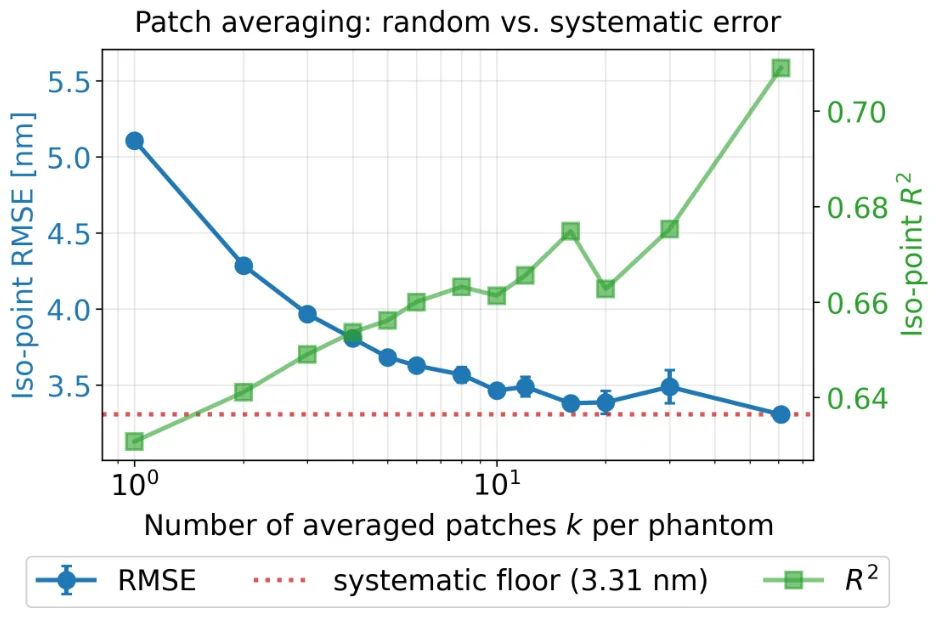
Decomposition of the per-patch iso-point error into an averageable (random) and a systematic component. The predictions of *k* patches of the same phantom are averaged (random partition into blocks of size *k*, Monte-Carlo over partitions; k=1 uses every patch individually and therefore reproduces the per-patch metrics), and the RMSE (blue, left axis) and $R^{2}$ (green, right axis) are re-computed at each *k* with the same definition as the headline metric. At *k* equal to all patches of a bore (one prediction per inclusion) the number of evaluation points equals the number of phantoms. RMSE falls from ≈5.0 nm to a plateau of 3.27 nm (red dotted line) while $R^{2}$ rises to ≈0.71: the decrease is the averageable patch-level noise; the plateau is the irreducible systematic per-phantom bias.

**Figure 5:**
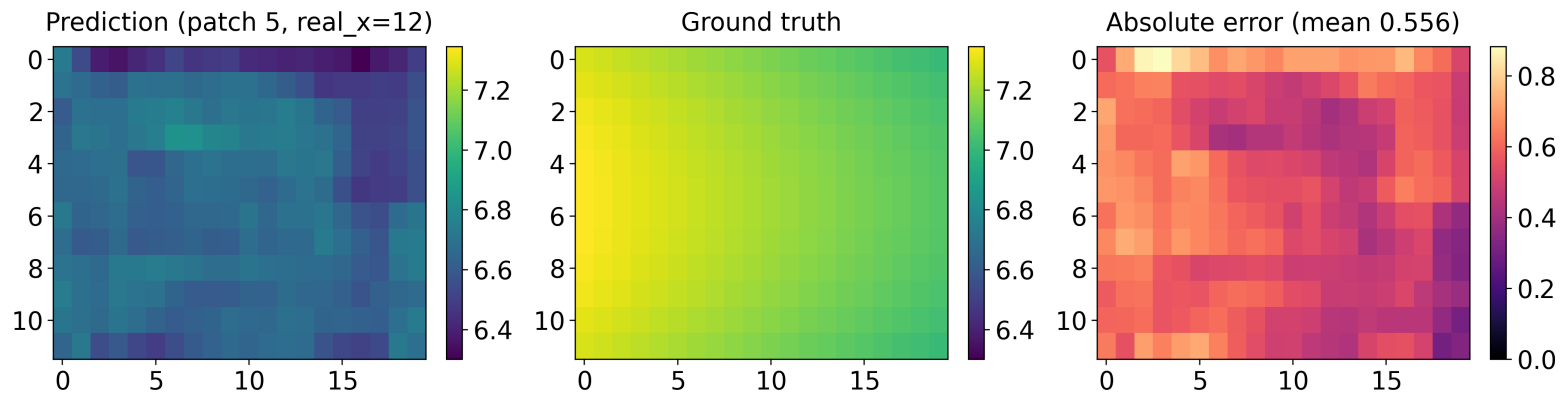
Representative inclusion depth map (one test patch, cropped to its $x_{\mathrm{real}}=12$ true pixels): prediction (**left**), ground truth (**middle**), and absolute error (**right**). The smooth left-to-right gradient in the ground truth is the depth variation along the oblique bore; the network reproduces it with a mean absolute error of ≈0.5 depth units.

**Figure 6:**
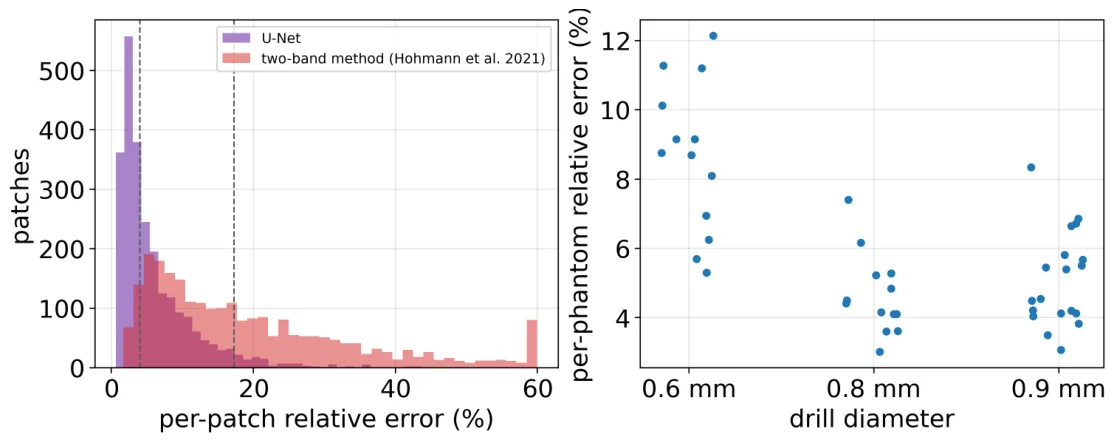
Distribution of the depth error over the test sets of all five seeds, from a re-run on the identical splits. **Left**: per-patch masked relative error of the U-Net (2488 patches) and of the validation-selected two-band method of reference [2]; dashed lines mark the median and the 95th percentile of the U-Net, and two-band errors above 60% are collected in the last bin. **Right**: per-phantom error of the U-Net (45 test phantoms) by drill diameter, with horizontal jitter.

**Figure 7:**
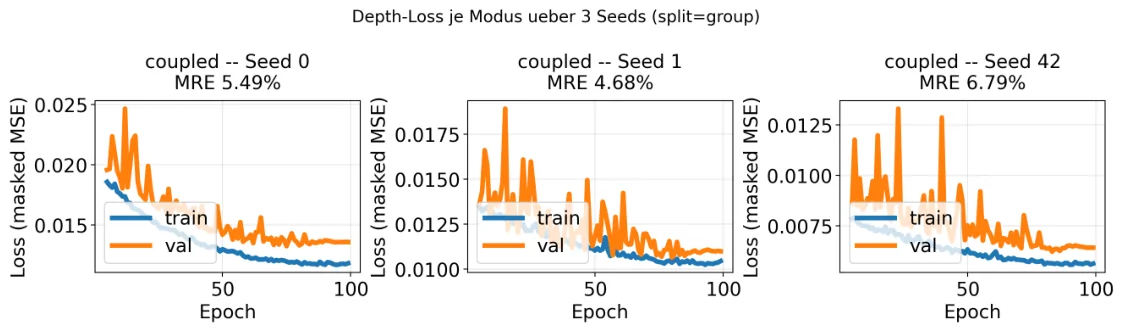
Depth training (blue) and validation (orange) loss across three seeds for the depth network conditioned on a jointly predicted iso-point via FiLM (group split). The per-seed masked mean relative error (titles) stays within the seed spread of the unconditioned baseline (6.07±0.71%); the oracle variant (true iso-point as the conditioning signal) behaves identically. Conditioning the depth reconstruction on the iso-point therefore yields no measurable benefit.

**Table 1:** Layers of the iso-point network (BiLT scanner, ≈32 k trainable parameters). The augmentation layer is active during training only. The input is the per-wavelength spatial-statistics sequence of shape (120,7) computed over the true (non-pad) pixels.

Layer	Type	Parameters
–	Spectral augmentation	max_shift =5, max_noise =0.025, ratio =0.5
*BiLT scanner*		
1	Conv1D	filters =32, kernel =15, GELU
2	MaxPooling1D	pool =3, stride =1
3	LayerNormalization	–
4	Positional embedding	120→32
5	Cross-attention	2 heads, key dim =32; 4 probes
6	LayerNormalization (+ residual)	–
7	Self-attention	2 heads, key dim =32
8	LayerNormalization (+ residual)	–
9	Dense (FFN) + LayerNorm (+ residual)	64→32, GELU
10	Flatten	4×32=128
*Regression head*		
11	Dense	32, GELU, L2 $=10^{-3}$
12	Dropout	0.4
13	Dense (output)	1, linear

**Table 2:** Layers of the depth-map network (masked residual U-Net). Each residual block is two k×k convolutions (batch normalisation and ReLU) with a 1×1 projection shortcut and stochastic-depth, fused by addition; all convolutions carry an L2 kernel penalty of $10^{-4}$. The decoder uses skip connections to the matching encoder stages ($c_{1}$, $c_{2}$). The input is the zero-padded patch cube of shape (20,20,120); the output is the dense depth map, evaluated through the binary real-pixel mask (Equation (1)).

Stage	Type	Parameters
–	Input	20×20×120
*Encoder*		
1a	Residual block (spatial)	48 filters, 3×3, survival =0.95
1b	Residual block (spectral)	48 filters, 1×1, survival =0.85
1	Add (fusion) + MaxPooling2D	→10×10×48
2	Residual block + MaxPooling2D	96 filters, 3×3, survival =0.9; →5×5×96
*Bottleneck*		
3	Conv2D + BN + ReLU	192 filters, 3×3
4	Conv2D + BN + ReLU	192 filters, 3×3
5	SpatialDropout2D	0.15
*Decoder*		
6	Conv2DTranspose + skip ($c_{2}$)	96 filters, stride 2; →10×10
7	Residual block	96 filters, 3×3, survival =0.95
8	UpSampling2D + skip ($c_{1}$)	→20×20
9	Residual block	48 filters, 3×3, survival =0.95
10	Conv2D (output)	1 filter, 1×1, linear

**Table 3:** Iso-point prediction under the cross-phantom group split (n=5 seeds, mean ± std). The spectrum-only model is the policy-compliant default. The oracle row is a diagnostic upper bound, not a deployable model. The bottom row quantifies the effect of the label-pipeline fix: the network is retrained on the labels of the earlier detector, with input and split unchanged, so that only the label differs. All rows use the current configuration (Huber loss, contiguous patch sampling) on the identical splits.

Configuration	RMSE [nm]	$R^{2}$
BiLT scanner, spectrum-only (default)	5.03±0.93	0.630±0.085
+ full concentration oracle (diagnostic)	4.52±0.90	0.703±0.065
Earlier detector (wavelet + polyfit labels)	7.20±1.13	0.477±0.106

**Table 4:** Detector parameter sweep. Labels of each setting (Savitzky–Golay window, order, MAD threshold) compared with the reference setting (bold), and the iso-point network retrained on them with fixed input and split (n=5 seeds, mean ± std). “Own” refers to the labels of the respective setting, “vs. ref.” to the reference labels. One measurement jumps to a secondary minimum at 551 nm, the lower edge of the search window, for window 13 and 17 at order 4 and for MAD 0.6; it is excluded from *r* and RMSD and lies in none of the test sets.

Window	Order	MAD	*r*	RMSD	RMSE	$R^{2}$	RMSE
			vs. Ref.	[nm]	Own [nm]	Own	vs. Ref. [nm]
13	4	0.8	0.74	7.40	7.73±1.33	0.44±0.19	5.51±0.59
17	4	0.8	0.86	4.99	6.95±1.29	0.43±0.20	5.26±0.97
23	2	0.8	0.90	3.74	4.99±0.80	0.68±0.08	5.22±0.94
23	3	0.8	0.90	3.74	4.95±0.90	0.69±0.08	5.13±0.90
23	4	0.6	0.999	0.27	5.08±0.88	0.62±0.09	5.06±0.88
**23**	**4**	**0.8**	1	0	4.84±0.85	0.66±0.06	4.84±0.85
23	4	1.0	0.999	0.32	4.95±0.67	0.64±0.06	4.92±0.72
31	4	0.8	0.91	3.62	5.33±0.58	0.60±0.06	5.18±0.93

## Data Availability

The processed data and the code that generates them are provided as Supplementary Materials. The raw hyperspectral cubes, 238 files of 825 MiB in total, are available on request from the author.

## Supplementary Materials

The following supporting information can be downloaded at https://www.mdpi.com/article/10.3390/s26206381/s1, The processed dataset and the analysis code are available as Supplementary Material. The archive holds the per-patch iso-point features and labels, the depth ground truth with its pixel masks, the measurement and phantom-configuration tables, a description of every variable with its unit, and the scripts that build the dataset, recompute the reported numbers and train the two networks. The code and the stored results of the analyses added in the revision are included as well, so their numbers recompute without the raw data.
